# Prevalence and risk factors of depression in Korean patients with untreated obstructive sleep apnea

**DOI:** 10.3389/fneur.2025.1643587

**Published:** 2025-08-29

**Authors:** Jeongsik Kim, Hea Ree Park, Eun Yeon Joo, Dae-Won Seo, Young-Min Shon

**Affiliations:** ^1^Department of Neurology, Neuroscience Center, Samsung Medical Center, Sungkyunkwan University School of Medicine, Seoul, Republic of Korea; ^2^Department of Medical Device Management and Research, Samsung Advanced Institute for Health Science and Technology, Sungkyunkwan University, Seoul, Republic of Korea; ^3^Smart Healthcare Research Institute, Samsung Medical Center, Seoul, Republic of Korea

**Keywords:** obstructive sleep apnea, depression, prevalence, risk factors, cognitive symptoms

## Abstract

**Introduction:**

This study investigated the prevalence and risk factors of depression among Korean adults with untreated obstructive sleep apnea (OSA).

**Methods:**

A total of 887 adults newly diagnosed with OSA at a university hospital underwent overnight polysomnography and completed validated Korean questionnaires assessing depression (BDI-II-K), insomnia (ISI-K), sleep quality (PSQI-K), and daytime sleepiness (ESS-K). Clinical depression was defined as a BDI-II-K score ≥17. Demographic, clinical, and sleep-related variables were compared between OSA patients with and without depression. Logistic regression identified independent risk factors, and factor analysis of the BDI-II-K explored symptom structure.

**Results:**

Clinical depression was present in 24.2% of OSA patients. The depression group had a higher proportion of women, older age, more frequent use of sleeping pills, and lower rates of regular exercise and employment. Polysomnography revealed longer sleep latency, lower sleep efficiency, and reduced N3 sleep in the depression group, but no difference in AHI severity or oxygen desaturation. Questionnaire data showed greater daytime sleepiness, more severe insomnia, and poorer sleep quality among depressed patients. Multivariable analysis identified higher arousal index (OR = 3.51), greater daytime sleepiness (OR = 1.36), and increased insomnia severity (OR = 5.27) as risk factors for depression, while AHI was not. Factor analysis indicated cognitive symptoms were most strongly associated with depression.

**Conclusion:**

Depression is common in Korean patients with untreated OSA, with sleep fragmentation, insomnia, and daytime sleepiness as key risk factors. Cognitive symptoms predominate the depressive profile. Routine depression screening and integrated management addressing both sleep and mood disturbances are recommended for this population.

## 1 Introduction

Obstructive sleep apnea causes sleep disruption and intermittent oxygen reduction, which impair daytime alertness, thinking processes, and emotional state. Among the biological mechanisms involved, serotonin participates in both mood control and upper airway regulation; decreased serotonergic function during sleep may worsen both OSA and depressive manifestations. Notably, the clinical presentation of OSA differs between males and females, with women more likely to report symptoms such as insomnia and depression, whereas men more often present with witnessed apneas and excessive daytime sleepiness (1).

Sleep quality significantly affects various aspects of daily functioning, including cognitive performance, mood regulation, and overall well-being. OSA is a major risk factor for reduced sleep quality, leading to fragmented sleep and daytime dysfunction (2).

Obesity is recognized as the most common and significant risk factor for obstructive sleep apnea (OSA), contributing to upper airway narrowing through increased adipose tissue around the pharyngeal region, which results in repeated episodes of airway obstruction during sleep (3). Research suggests that sleep fragmentation, rather than reduced oxygen levels, constitutes the primary connection between OSA and depression. Recurrent nighttime awakenings disrupt slow-wave and REM sleep, essential for emotional and cognitive functioning, and chronic sleep disruption can modify frontal brain activity, increasing vulnerability to mood disturbances even without clinical depression (4).

The frequency of depression among OSA patients varies extensively across studies, with rates spanning from 5 to 63% (5) contingent upon assessment techniques, population characteristics, and diagnostic criteria. Elevated rates have been observed both in general populations (6) and in specific groups such as US veterans (7). Korean studies utilizing the Beck Depression Inventory (BDI ≥ 10) reported prevalence between 39 and 48.4% (8, 9). More meta-analyses, however, suggest a combined prevalence of 23–35% for clinically significant depressive manifestations in OSA patients (10). These variations are influenced by demographic elements including gender and age, clinical features such as daytime sleepiness and sleep quality, and differences in depression assessment tools. Notably, women with OSA frequently report higher rates of depressive symptoms and present with sleep difficulties, fatigue, and mood alterations rather than classic OSA manifestations, contributing to diagnostic delays and prevalence differences across studies with varied gender compositions (10).

Although the BDI-II is widely used for evaluating depression in OSA patients, there are concerns about its diagnostic accuracy because of the overlap between OSA and depression symptoms, as it includes somatic symptoms (e.g., fatigue, sleep disturbances). This symptom convergence may artificially elevate BDI-II scores, leading to under-diagnosis of OSA in depressed patients and under-recognition of depression in OSA cases (11). The bidirectional relationship creates a cyclical pattern in which sleep disturbance worsens depressive symptoms, which in turn further reduces sleep quality. Factor analysis of the BDI-II domains (cognitive, affective, and somatic) can help to differentiate OSA-related physical symptoms from genuine depressive comorbidity, allowing for tailored interventions for each symptom clusters.

This study investigates depression prevalence and risk factors in 887 untreated Korean OSA patients using more stringent criteria (BDI-II-K ≥ 17) than prior research. Through a mixed-gender cohort, it addresses limitations of earlier male-dominated studies. The methodology combines objective sleep monitoring (assessing sleep architecture, arousal index, AHI) with validated Korean questionnaires (BDI-II-K, ISI-K, PSQI-K, ESS-K) to evaluate subjective sleep quality, insomnia, and daytime sleepiness. Multivariable logistic regression identifies independent depression risk factors while accounting for confounding variables such as age, sex, and medication use. The analysis specifically examines whether sleep fragmentation, reduced oxygen levels, or subjective complaints show stronger depression associations. Additionally, factor analysis of BDI-II-K domains (cognitive, affective, and somatic) clarifies whether elevated scores reflect true mood disorders or OSA-related somatic symptoms. This comprehensive approach aims to untangle OSA-depression comorbidity mechanisms and enhance diagnostic precision in clinical practice.

## 2 Methods

### 2.1 Subjects

This study was approved and conducted by Institutional Review Board. All participants underwent a full-night polysomnography (PSG) and a set of sleep-related questionnaires including the Korean versions of the BDI-II (BDI-II-K), the Insomnia Severity Index (ISI-K), the Pittsburg Sleep Quality Index (PSQI-K), and the Epworth Sleepiness Scale (ESS-K) at the Samsung Medical Center sleep center between May 2018 and February 2020. Based on PSG and clinical findings, 887 individuals were diagnosed with OSA and enrolled in this study. No participants had prior history of treatment for OSA or intake of antidepressant medication before this study. Inclusion criteria encompassed ages 18–88 years and an Apnea-Hypopnea Index (AHI) of ≥5 events/h. Individuals who had comorbid disorders other than insomnia (i.e., restless legs syndrome, narcolepsy, parasomnia, and epilepsy), and those with cognitive impairment severe enough to interfere with their ability to participate were excluded from the study.

### 2.2 Overnight polysomnography

PSG is the gold standard for diagnosing OSA and evaluating its severity, providing comprehensive, objective measurement of multiple physiological parameters including respiratory events, sleep stages, arousals, and oxygen saturation, which are essential for accurate assessment and personalized management of the disorder (12). OSA is diagnosed when overnight PSG demonstrates an AHI of 5 or more events per hour accompanied by relevant symptoms such as excessive daytime sleepiness, or an AHI of at least 15 events per hour regardless of symptoms, according to internationally accepted guidelines (13).

All patients were asked to abstain from alcohol or caffeinated beverages on the day before the sleep studies were conducted. Sleep studies were recorded using a Somnologica (Embla; Denver, CO, United States). PSG was recorded using a six-channel electroencephalogram (F3/A2; F4/A1; C3/A2; C4/A1; O1/A2; O2/A1), a four channel electrooculogram, an electromyogram (on submental, intercostal, and anterior tibialis muscles), and an electrocardiogram with surface electrodes. Subjects were also attached with a thermistor (for monitoring oronasal airflow), nasal air pressure monitor, an oximeter (for measuring oxygen saturation), piezoelectric bands (for determining thoracic and abdominal wall motion), and a body-position sensor. Subjects went to bed at 23:00 and were awakened at 07:00 the next day. Sleep architecture was scored in 30-s epochs, and sleep staging was interpreted in accordance with the criteria of Rechtschaffen and Kales. Apneas and hypopneas were defined by standard scoring scales. The American Academy of Sleep Medicine (AASM) rules (AASM manual version 2.0) were used for scoring hypopnea as follows: more than 30% reduction in nasal pressure signal excursions from baseline that lasted more than 10 s with a more than 3% desaturation from the pre-event baseline or arousal. Apnea was defined as a reduction in airflow >90% in thermistor lasting ≥10 s during which there was evidence of persistent respiratory effort. To grade the severity of sleep apnea, the number of apnea and hypopnea events per hour was reported as the apnea-hypopnea index (AHI). OSA severity was defined as follows: (AHI < 5/h, normal; AHI 5-15/h, mild; AHI 15–30, moderate; AHI > 30, severe).

### 2.3 Assessments of sleep-related and clinical depression questionnaires

Depression is a common mood disorder marked by persistent sadness, loss of interest, and impaired daily functioning. It is frequently assessed in clinical and research settings using validated self-report questionnaires such as the Beck Depression Inventory (BDI) and the Patient Health Questionnaire-9 (PHQ-9), which help quantify symptom severity and facilitate diagnosis (14). In this study, all subjects completed four questionnaires to assess depression (BDI-II-K) (15), excessive daytime sleepiness (EDS) (ESS-K) (16), insomnia severity (ISI-K) (17), and sleep quality (PSQI-K) (18). In our study, to evaluate the prevalence of clinical depression (depressive-related disorder) in our subjects, the 17 points score of BDI-II-K was used as a cut-off value to detect clinical depression in OSA patients (19). All subjects were divided into two groups based on their scores on the BDI-II-K: OSA with depression (OSA + D) (BDI-II-K score ≥ 17) and OSA without depression (OSA − D) (BDI-II-K score < 17). We also obtained participants’ BDI-II sub-factor scores using the Buckley three-factor model, which is composed by cognitive, affective and somatic symptoms (20), to determine whether the depressive symptoms in OSA patients were related to somatic, cognitive or affective symptoms.

### 2.4 Statistical analysis

Differences between OSA + D and OSA − D groups in demographic, clinical, PSG and sleep questionnaires’ variables were tested using the Wilcoxon rank-sum test or *t*-test for continuous variables and using the Pearson’s Chi-square test or Fisher’s exact test for categorical variables. The univariable and multivariable logistic regression models were applied to find the risk factors of sleep-related variables for depression between the two groups. The clinically and statistically significant factors including age, sex, presence of insomnia, and use of sleeping pills were adjusted in multivariable models. The odds ratio (OR) and 95% confidence interval (CI) were presented as the results. In addition, in order to more deeply examine the effect of sleep apnea on depression, we analyzed the difference in the incidence of depression according to the severity of sleep apnea (graded depending on the AHI. mild OSA: AHI of 5 to <15; moderate OSA: AHI of 15 to 30; severe OSA: AHI ≥ 30). The results were considered statistically significant when two-sided *p*-values were less than 0.05. To perform the factor analysis of BDI-II, each factor of the Berkley model was converted into 100 points, and used to the univariable regression analysis. All analyses were performed using SAS software, version 9.4 (SAS Institute Inc., Cary, NC, United States.).

## 3 Results

The mean age was 51.6 ± 13.5 years, mean BMI 27.0 ± 4.4 kg/m^2^, mean AHI 36.9 ± 23.9/h, and 143 [16.1%] women. Of the 887 subjects, 215 subjects (24.2%) had clinically significant depression. The median BDI-II-K scores in OSA + D and in OSA − D groups were 20 [interquartile range (IQR), 18–26] and 8 [IQR, 5–12], respectively. As a result of univariable logistic regression analysis of the BDI-II-K sub-factor scores in OSA + D group, cognitive factor showed the highest risk of developing depression (OR = 13.77, *p* < 0.001) compared to somatic (OR = 7.91, *p* < 0.001) and affective factor (OR = 5.72, *p* < 0.001).

Differences in demographic and clinical characteristics between OSA + D and OSA − D groups were presented in Table 1. The OSA + D group had a higher percentage of women than the OSA − D group (23.7% versus 13.7%, *p* < 0.001). Among the demographic and clinical variables, OSA + D group had a higher age (*p* = 0.009), a lower rate of regular exercise (*p* = 0.003), a lower rate of occupation (*p* = 0.021), and a higher rate of taking sleeping pills (*p* < 0.001) compared to the OSA − D group. However, there was no difference between the groups in comorbidities.

**Table 1:** Demographic and clinical characteristics between OSA patients with and without depression.

Characteristics	Total (*n* = 887)	OSA + D (*n* = 215)	OSA − D (*n* = 672)	*p*-value
Demographics				
Sex, *n* (%)				
Men, *n* (%)	744	164 (22)	580 (78)	0.001
Women, *n* (%)	143	51 (35.7)	92 (64.3)	
Age, mean (SD), year	51.6 (13.5)	53.1 (13.8)	51.2 (13.4)	0.064
Age < 45, *n* (%)	256	57 (22.3)	199 (77.7)	0.009
Age 45 to 64, *n* (%)	488	109 (22.3)	379 (77.7)	
Age > 64, *n* (%)	143	49 (34.3)	94 (65.7)	
Body mass index, mean (SD), kg/m^2^	27.0 (4.4)	27.5 (5.3)	26.9 (4.0)	0.102
Job, *n* (%)				
Unemployed	187	58 (31)	129 (69)	0.021
Student	27	9 (33.3)	18 (66.7)	
Employed	673	148 (22)	525 (78)	
Smoking, *n* (%)				
Non-smoker	520	122 (23.5)	398 (76.5)	0.280
Smoker	162	47 (29)	115 (71)	
Stopped smoking	205	46 (22.4)	159 (77.6)	
Caffeine, *n* (%)				
Yes	765	181 (23.7)	584 (76.3)	0.314
No	122	34 (27.9)	88 (72.1)	
Exercise, *n* (%)				
Yes	411	81 (19.7)	330 (80.3)	0.003
No	476	134 (28.2)	342 (71.9)	
Clinical				
Comorbidity-hypertension, *n* (%)				
Yes	305	76 (24.9)	229 (75.1)	0.733
No	582	139 (23.9)	443 (76.1)	
Comorbidity-diabetes, *n* (%)				
Yes	115	35 (30.4)	80 (69.6)	0.097
No	772	180 (23.3)	592 (76.7)	
Comorbidity-heart disease, *n* (%)				
Yes	67	19 (28.4)	48 (71.6)	0.413
No	820	196 (23.9)	624 (76.1)	
Comorbidity-hyperlipidemia, *n* (%)				
Yes	39	9 (23.1)	30 (76.9)	0.863
No	848	206 (24.3)	642 (75.7)	
Comorbidity-vascular disease, *n* (%)				
Yes	3	1 (33.3)	2 (66.7)	0.566
No	884	214 (24.2)	670 (75.8)	
Use of sleeping pills, *n* (%)				
Yes	56	32 (57.1)	24 (42.9)	<0.001
No	831	183 (22)	648 (78)	

The results of comparing the sleep-related variables between OSA + D and OSA − D groups are summarized in Table 2. Subjects in the OSA + D group had longer sleep latency (6.5 min versus 5.5 min, *p =* 0.008), longer REM sleep latency (93.5 min versus 85.8 min, *p* = 0.006), lower sleep efficiency (83.9% versus 86.4%, *p* = 0.038), and lower percentage of stage N3 sleep (0.8% versus 1.3%, *p* = 0.047). However, there was no significant difference between the two groups in severity of AHI and SaO2 nadir. Among the sleep questionnaires, subjects in the OSA + D group were more daytime sleepiness (ESS scores: 10.0 versus 9.0, *p* < 0.001), more insomnia severity (ISI scores: 13.0 versus 8.0, *p <* 0.001), and lower subjective sleep quality (PSQI scores: 8.0 versus 6.0, *p* < 0.001) than those in the OSA − D group.

**Table 2:** Polysomnography and sleep questionnaires between OSA patients with and without depression.

Variables	Total (*n* = 887)	OSA + D (*n* = 215)	OSA − D (*n* = 672)	*p-*value
Polysomnography				
Total sleep time, min	361.5 [315, 400]	353 [295.5, 396]	363.3 [318.8, 403.8]	0.074
Sleep latency, min	5.5 [3, 11.5]	6.5 [3, 14]	5.5 [2.8, 10.5]	0.008
REM sleep latency, min	87 [64, 129.5]	93.5 [70, 142.5]	85.8 [62.5, 125.5]	0.006
Wakefulness after sleep onset, min	51 [30, 82.5]	54 [33, 87]	49.8 [29.2, 81]	0.076
Wakefulness after sleep onset, %	12.4 [7.3, 20.3]	13.9 [8, 21.5]	12 [7.2, 19.7]	0.050
Sleep efficiency, % TST	85.7 [77.7, 91.3]	83.9 [76.2, 90.7]	86.4 [78.3, 91.5]	0.038
Stage N1 sleep, % TST	21.4 [14.5, 31.1]	22.2 [15.4, 31]	21.1 [14, 31.1]	0.290
Stage N2 sleep, % TST	53.5 [45.5, 60]	54.7 [46.9, 61]	53 [45.1, 59.7]	0.101
Stage N3 sleep, % TST	1.2 [0, 7.2]	0.8 [0, 5.5]	1.3 [0, 8]	0.047
Stage REM sleep, % TST	18.9 [14.3, 23]	18.3 [14.1, 22.8]	19.1 [14.5, 23.3]	0.129
Arousal index, events/h	26.6 [18.3, 37.9]	28.6 [19.3, 39.9]	26.3 [18, 37.5]	0.064
AHI, events/h	32.3 [17.6, 51.8]	32.4 [18.3, 53]	32.3 [17.2, 51.4]	0.186
SaO2 nadir, %	83 [77, 87]	84 [78, 87]	83 [77, 87]	0.699
Oxygen desaturation index, events/h	25.1 [12.8, 44.9]	25.7 [14, 49.2]	24.7 [12.6, 44.7]	0.303
Questionnaires				
Epworth sleepiness scale	9 [6, 13]	10 [7, 15]	9 [6, 12]	<0.001
Beck depression inventory-II	10 [6, 16]	20 [18, 26]	8 [5, 12]	<0.001
Insomnia severity index	9 [5, 14]	13 [11, 17]	8 [4, 12]	<0.001
Pittsburg sleep quality index	6 [4, 9]	8 [6, 11]	6 [4, 8]	<0.001

Summary statistics were presented as median and interquartile range [IQR].

After adjusting for confounding variables (age, sex, presence of insomnia, and use of sleeping pills) which showed significant differences between OSA + D and OSA − D groups, multivariable analyses of the variables in the PSG and the sleep questionnaires showed that higher arousal index (OR = 3.51, 95% CI [1.17, 10.54]), higher ESS scores (OR = 1.36, 95% CI [0.97, 1.91], *p* < 0.042) and higher ISI scores (OR = 5.27, 95% CI [3.36, 8.25], *p* < 0.001) were risk factors of developing depression (Tables 3, 4).

**Table 3:** Univariable and multivariable logistic regression models for polysomnography.

Variable	OSA + D (*n* = 215)	OSA − D (*n* = 672)	Univariable model*		Multivariable model**	
			OR (95% CI)	*p-*value	OR (95% CI)	*p-*value
Total sleep time, 10 min			0.98 (0.96, 1)	0.0929	0.99 (0.96, 1.01)	0.243
Sleep latency, min						
<10	139 (22.3)	485 (77.7)	1 (ref)	(0.0309)	1 (ref)	(0.149)
10 to 20	44 (25.7)	127 (74.3)	1.21 (0.82, 1.79)	0.3421	1.08 (0.72, 1.61)	0.745
>20	32 (34.8)	60 (65.2)	1.86 (1.17, 2.97)	0.0094	1.61 (0.98, 2.62)	0.051
REM sleep latency, min						
<80	74 (19.5)	305 (80.5)	1 (ref)	(0.0187)	1 (ref)	(0.015)
80 to 120	69 (27.5)	182 (72.5)	1.56 (1.07, 2.28)	0.02	1.77 (1.18, 2.64)	0.006
>120	72 (28)	185 (72)	1.6 (1.11, 2.33)	0.0129	1.51 (1.01, 2.24)	0.044
Wakefulness after sleep onset, 10 min			1.02 (0.99, 1.05)	0.1846	1.01 (0.97, 1.04)	0.664
Wakefulness after sleep onset, 10%			1.1 (0.97, 1.25)	0.1436	1.04 (0.90, 1.20)	0.589
Sleep efficiency, 10% TST			0.9 (0.8, 1.02)	0.1005	0.96 (0.83, 1.10)	0.539
Stage N1 sleep, % TST						
<5	1 (10)	9 (90)	1 (ref)	(0.5887)	1 (ref)	(0.447)
5 to 10	23 (25.3)	68 (74.7)	3.04 (0.37, 25.33)	0.3033	3.94 (0.43, 36.08)	0.226
>10	191 (24.3)	595 (75.7)	2.89 (0.36, 22.94)	0.3158	3.31 (0.38, 28.84)	0.279
Stage N2 sleep, % TST						
<45	45 (21.3)	166 (78.7)	1 (ref)	(0.3691)	1 (ref)	(0.582)
45 to 55	68 (23.5)	221 (76.5)	1.14 (0.74, 1.74)	0.5611	1.09 (0.69, 1.71)	0.725
>55	102 (26.4)	285 (73.6)	1.32 (0.89, 1.97)	0.173	1.24 (0.81, 1.89)	0.326
Stage N3 sleep, % TST						
<10	190 (26)	542 (74)	3.62 (1.09–11.97)	0.0351	3.03 (0.88, 10.41)	0.079
10 to 20	22 (18.2)	99 (81.8)	2.29 (0.64–8.18)	0.2005	2.07 (0.56, 7.65)	0.275
>20	3 (8.8)	31 (91.2)	1 (ref)	(0.0245)	1 (ref)	(0.093)
Stage REM sleep, % TST						
<18	100 (26.5)	277 (73.5)	1 (ref)	(0.3485)	1 (ref)	(0.212)
18 to 23	68 (23.4)	223 (76.6)	0.85 (0.59, 1.21)	0.3513	0.88 (0.60, 1.29)	0.509
>23	47 (21.5)	172 (78.5)	0.76 (0.51, 1.12)	0.1675	0.68 (0.45, 1.04)	0.078
Arousal index, events/h						
<10	4 (10.5)	34 (89.5)	1 (ref)	(0.0786)	1 (ref)	(0.040)
10 to 25	84 (22.9)	283 (77.1)	2.52 (0.87, 7.31)	0.0884	2.72 (0.90, 8.20)	0.076
>25	127 (26.4)	355 (73.7)	3.04 (1.06, 8.74)	0.039	3.51 (1.17, 10.54)	0.025
AHI, events/h						
Mild (AHI < 15)	30 (19.2)	126 (80.8)	1 (ref)	(0.2773)	1 (ref)	(0.241)
Moderate (15 < AHI < 30)	67 (25.4)	197 (74.6)	1.43 (0.88, 2.32)	0.1499	1.36 (0.82, 2.28)	0.237
Severe (AHI ≥ 30)	118 (25.3)	349 (74.7)	1.42 (0.91, 2.23)	0.1264	1.51 (0.94, 2.43)	0.092
SaO2 nadir, %						
Mild (≥90)	27 (27.6)	71 (72.5)	1.27 (0.76, 2.15)	0.3637	1.08 (0.63, 1.88)	0.773
Moderate (80 to 89)	122 (24.3)	380 (75.7)	1.08 (0.76, 1.51)	0.6787	0.97 (0.67, 1.41)	0.888
Severe (<80)	66 (23.0)	221 (77.0)	1 (ref)	(0.6616)	1 (ref)	(0.920)
Oxygen desaturation index, events/h						
<5	12 (23.1)	40 (76.9)	1 (ref)		1 (ref)	
≥5	203 (24.3)	632 (75.7)	1.07 (0.55, 2.08)	0.8403	1.20 (0.55, 2.08)	0.602

*Logistic regression model, OR, odds ratio; CI, confidence interval.

**Adjusted age, sex, presence of insomnia (ISI ≥ 8 vs. ISI < 8) and use of sleeping pills.

SaO2 nadir (%) variable: use Firth option.

**Table 4:** Univariable and multivariable logistic regression models for sleep questionnaires.

Variable		OSA + D (*n* = 215)	OSA − D (*n* = 672)	Univariable model*		Multivariable model**	
				OR (95% CI)	*p-*value	OR (95% CI)	*p-*value
Epworth sleepiness scale (ESS)	No EDS (ESS < 11)	111 (20.8)	424 (79.3)	1 (ref)		1 (ref)	
	EDS (ESS ≥ 11)	104 (29.6)	248 (70.5)	1.6 (1.18, 2.18)	0.003	1.36 (0.97, 1.91)	0.042
Insomnia severity index (ISI)	No insomnia (ISI < 8)	25 (7.9)	291 (92.1)	1 (ref)		1 (ref)	
	Clinical insomnia (ISI ≥ 8)	190 (33.3)	381 (66.7)	5.81 (3.72, 9.05)	<0.001	5.27 (3.36, 8.25)	<0.001
Pittsburg sleep quality index (PSQI)	PSQI < 5	26 (10.3)	226 (89.7)	1 (ref)		1 (ref)	
	PSQI ≥ 5	189 (29.8)	446 (70.2)	3.68 (2.37, 5.72)	<0.001	1.53 (0.92, 2.54)	0.099

EDS, excessive daytime sleepiness.

*Logistic regression model, OR: odds ratio, CI: confidence interval.

**Adjusted age, sex, presence of insomnia and use of sleeping pills.

## 4 Discussion

This study examined the prevalence of depression and its associated risk factors in patients with untreated OSA. Our findings showed that 24.2% of OSA patients exhibited clinically significant depression according to BDI-II-K scores, which aligns with previous research reporting prevalence rates ranging from 21 to 35%. The factor analysis of BDI-II-K demonstrated that cognitive symptoms showed the strongest association with depression in OSA patients, indicating that the cognitive component of depression might be particularly relevant in this population. Multivariable analysis identified higher arousal index, greater daytime sleepiness (ESS scores), and increased insomnia severity (ISI scores) as independent risk factors for depression in OSA patients after adjusting for potential confounders including age, sex, presence of insomnia, and use of sleeping pills. Interestingly, we discovered no significant connection between AHI severity and depression, suggesting that subjective symptoms and sleep fragmentation rather than the severity of respiratory events might play more crucial roles in the development of depressive symptoms in OSA patients.

### 4.1 Prevalence of depression in OSA

The observed 24.2% depression rate in our OSA cohort corresponds to prior meta-analytic findings reporting a 23% aggregated prevalence across seven studies (4, 5, 21–26), as well as contemporary studies documenting rates of 21–35% (10). These findings reside within the broader literature-reported spectrum of 5–63%, with discrepancies potentially stemming from methodological variations in assessment protocols, population demographics, and diagnostic criteria (10). This substantial prevalence highlights OSA’s considerable mental health implications.

Marked disparities emerged between genders regarding depression rates, with 23.7% of female OSA patients exhibiting depressive symptoms versus 13.7% of males-a pattern consistent with earlier research demonstrating elevated HADS-D scores among women with OSA (27). This divergence may stem from biological factors like hormonal fluctuations, distinct clinical symptom profiles, or gender-specific psychosocial responses to chronic health conditions. Extended analysis further reveals amplified depression rates in postmenopausal women (≥55 years) with OSA compared to age-matched male counterparts (28).

The high depression frequency in our sample underscores the critical need for clinical recognition of this comorbidity. While our results parallel international reports, the persistent association across heterogeneous populations implies that OSA-depression linkages maintain consistency across diverse cultural contexts.

### 4.2 Risk factors for depression in OSA

Multivariable regression modeling revealed three independent predictors of depression comorbidity: elevated arousal frequency (OR = 3.51), heightened daytime somnolence (ESS-adjusted OR = 1.36), and pronounced insomnia severity (ISI-derived OR = 5.27). These associations illuminate potential pathophysiological interactions between OSA and affective disturbances.

The robust correlation between arousal indices and depression implies that sleep continuity disruption, rather than nocturnal hypoxemia, serves as a principal mediator linking respiratory disturbances to mood disorders. Frequent cortical arousals impair sleep architecture consolidation, potentially hindering neural restoration processes critical for emotional regulation. This observation corroborates prior evidence demonstrating strong ties between sleep fragmentation metrics and cognitive deficits in OSA populations (29).

Excessive daytime somnolence emerged as a secondary predictive element, aligning with LaGrotte et al.’s identification of EDS as a depression precursor in OSA (30, 41). This relationship may exhibit bidirectionality, as depression-related anergia could exacerbate perceived sleepiness, while chronic somnolence may amplify affective symptoms through functional impairment. Our data further substantiate this interplay through marginal sleep quality-EDS linkages (*p* = 0.051) (30).

Insomnia severity demonstrated the strongest depression association, consistent with COMISA research showing amplified affective symptoms in dual-diagnosis patients versus isolated OSA (31). Lichstein et al.’s comparative analysis of COMISA cohorts revealed significantly elevated depression scores relative to OSA-only groups, underscoring insomnia’s critical role in mood pathogenesis (31, 42). These findings advocate for therapeutic strategies addressing sleep maintenance beyond conventional apnea management.

Notably, our analysis detected no significant AHI-depression correlation, contrasting with linear models proposing 0.4% depression risk escalation per AHI unit (32). Discrepancies may stem from methodological variations across studies, including: first, heterogeneous depression assessment tools (BDI vs. HADS vs. PHQ-9) yielding prevalence estimates ranging 5–63% (31, 33). Second, our cohort’s exclusive focus on treatment-naïve Korean patients with rigorous confounder control (age, sex, hypnotic use). Third, dominant influence of subjective sleep perceptions over polysomnographic respiratory metrics (33, 34). Specifically, our multivariable analysis identified higher arousal index (OR = 3.51), greater daytime sleepiness (ESS, OR = 1.36), and more severe insomnia (ISI, OR = 5.27) as independent risk factors for depression, while AHI severity showed no significant association. This suggests that the psychological impact of OSA may be more closely linked to sleep disruption and its daytime consequences than to the frequency of apneic events themselves, highlighting the complex, multifactorial nature of the OSA-depression relationship (9, 34).

Our findings indicate that insomnia severity and increased sleep arousals are significant risk factors for depression in OSA patients, highlighting the importance of sleep continuity and fragmentation in mood regulation. Although sleep efficiency showed a trend toward association with depression, it did not reach statistical significance in our cohort. This may reflect the complex interplay between subjective sleep complaints and objective sleep measures, where insomnia symptoms and frequent micro-arousals more directly impact emotional and cognitive functioning than the global measure of sleep efficiency. Moreover, sleep efficiency can be influenced by various external factors and may not capture transient disruptions that contribute to depressive symptoms. Therefore, focusing on insomnia severity and arousal frequency provides a more sensitive gauge of sleep-related depression risk in this population.

In addition to well-known risk factors, recent studies indicate that dental malocclusions—such as overbite, underbite, and crossbite—may worsen obstructive sleep apnea (OSA) by narrowing the upper airway during sleep (35, 36). Additionally, certain congenital craniofacial conditions, including Treacher Collins syndrome, are important contributors to OSA risk due to structural airway abnormalities (37). These findings underscore the need for thorough evaluation of dental occlusion and craniofacial anomalies when assessing OSA risk.

### 4.3 Factor analysis of BDI-II in OSA

BDI-II-K factor analysis demonstrated cognitive manifestations as the most strongly correlated with depression in OSA cohorts (OR = 13.77), surpassing somatic (OR = 7.91) and affective (OR = 5.72) components. This counters prevalent assumptions that OSA-related physical symptoms artificially elevate BDI scores through diagnostic overlap.

The cognitive symptom predominance corresponds to established BDI-II dimensionality comprising cognitive-affective and somatic-vegetative domains (38). This pattern indicates OSA-associated depression extends beyond sleep-related physical complaints, involving substantive cognitive disturbances including persistent self-critical ideation and negative future outlooks.

Neurobiological mechanisms may underpin this cognitive emphasis, as recurrent hypoxic episodes and sleep architecture disruption in OSA impair prefrontal regulatory circuits governing emotional processing (21). Furthermore, persistent OSA manifestations may foster maladaptive cognitive patterns akin to learned helplessness models, where chronic sleep dysfunction reinforces pessimistic appraisals of daytime functioning.

Clinically, these findings argue for depression assessment protocols emphasizing cognitive markers (e.g., guilt perceptions, concentration difficulties) alongside traditional somatic indicators in OSA populations. Therapeutic approaches incorporating cognitive-behavioral strategies targeting dysfunctional thought patterns may prove particularly efficacious for mood management in this comorbidity context.

### 4.4 Notable differences from previous Korean studies

The present study demonstrates significant divergences from earlier Korean studies examining OSA-depression interactions. By utilizing more rigorous diagnostic thresholds (BDI-II-K ≥ 17 versus historical BDI ≥ 10 criteria), this work documents reduced depression prevalence (24.2% versus 39–48.4%), highlighting the necessity for uniform assessment protocols (8, 9). Contrary to established mediated apnea-depression models and inverse severity gradients, our analysis revealed no significant associations between AHI/oxygen metrics and depression (8, 9, 39). The identification of previously unreported risk markers (arousal index OR = 3.51; insomnia OR = 5.27) contrasts with former investigations prioritizing sleep quality metrics and gender-specific REM-related respiratory patterns (8, 9). Novel risk factors emerged (arousal index OR = 3.51, insomnia OR = 5.27), differing from prior emphasis on sleep quality and male-specific REM-breathing associations (8, 9, 39, 40). The observed female predilection for depressive comorbidity (35.7% vs. 22% males) contests conventional pathophysiological models centered on male populations (40). Notable methodological advancements encompass demonstrating cognitive symptom dominance in depression evaluation (OR = 13.77 vs. 7.91 somatic) and generating foundational data through treatment-naïve cohort analysis. These findings enhance mechanistic understanding while exposing methodological heterogeneities requiring harmonized research paradigms for robust comparative analyses.

### 4.5 Limitations

This study has several methodological limitations that should be acknowledged. The cross-sectional design prevents establishing causal relationships between OSA and depression, highlighting the need for longitudinal studies to clarify the directionality. The reliance on self-report measures introduces potential recall bias, although the validated reliability of the BDI-II somewhat mitigates this concern. The absence of a non-OSA control group limits the ability to compare prevalence rates directly, and unmeasured confounding variables such as socioeconomic factors may influence the observed associations. Additionally, recruiting participants from a clinical setting may overrepresent severe cases, which could reduce the generalizability of the findings. Finally, the lack of analysis on the temporal relationship between OSA onset and depression development underscores the need for future research to determine the sequence of these conditions.

### 4.6 Clinical implications

Previous studies have primarily focused on apnea severity as a marker for depression risk in OSA patients. However, our findings suggest that subjective sleep disturbances and sleep fragmentation contribute significantly to depression, independent of apnea severity.

Our study shows that sleep fragmentation, insomnia severity, and daytime sleepiness are closely linked to depression in untreated OSA patients, independently of apnea severity. This novel focus on subjective sleep disturbances enhances understanding of OSA-depression comorbidity beyond traditional respiratory measures. Using a large, well-characterized Korean cohort with detailed polysomnography and validated questionnaires adds strength and clinical relevance to our findings. These results highlight the need for integrated clinical screening of depression in OSA and support treatments that improve sleep quality, such as cognitive-behavioral therapy and optimized OSA management, to reduce depression risk and improve patient outcomes through a multidisciplinary approach.

The 24.2% depression prevalence in OSA patients underscores the necessity for systematic affective screening, particularly among women and those presenting with insomnia or excessive daytime somnolence. Factor analysis revealed cognitive depressive manifestations (e.g., pervasive negativity, anhedonia) as predominant markers, advocating for screening protocols prioritizing these over somatic symptoms. The robust insomnia-depression association (OR = 5.27) highlights sleep continuity management as a critical OSA care component, with CBT-I showing particular promise for comorbid cases. Clinicians should note that subjective daytime impairment-independent of AHI severity-correlates with depression risk, warranting targeted alertness interventions.

These results emphasize the importance of a comprehensive clinical assessment that integrates mood evaluation alongside sleep disorder parameters for more effective management of OSA patients. Considering both subjective symptoms and objective sleep measurements may optimize personalized treatment strategies, potentially improving long-term mental health and quality of life in this population.

## 5 Conclusion

This study underscores the considerable psychological burden imposed by depression comorbidity in obstructive sleep apnea (OSA) populations, with critical risk determinants encompassing sleep continuity disruption, pronounced daytime fatigue, and clinically significant insomnia. The predominance of cognitive-affective symptoms within depression profiles necessitates enhanced screening protocols prioritizing thought patterns (e.g., persistent negativity, self-devaluation) alongside traditional somatic indicators. Optimal therapeutic outcomes may require integrative strategies concurrently addressing respiratory pathophysiology and neurobehavioral components, emphasizing bidirectional management of sleep architecture restoration and mood stabilization.

## Data Availability

The raw data supporting the conclusions of this article will be made available by the authors upon reasonable request and subject to institutional review board approval.
