# Supplementary material for: Prevalence and risk factors of depression in Korean patients with untreated obstructive sleep apnea
Source: Front Neurol. 2025 Aug 29;16:1643587. doi: 10.3389/fneur.2025.1643587 (PMC12447800; doi:10.3389/fneur.2025.1643587)
Supplement: Supplementary file 1 [file Data_Sheet_1.docx]

**Supplementary Material: Assessment Instruments**

**1. Korean Version of Epworth Sleepiness Scale (ESS)**

Example Items:

앉아서 책(신문, 잡지, 서류 등)을 읽을 때

0 = 전혀 졸리지 않다

1 = 약간 졸리다

2 = 종종 졸리다

3 = 자주 졸리다

공공장소(모임, 극장 등)에서 가만히 앉아있을 때

0 = 전혀 졸리지 않다

1 = 약간 졸리다

2 = 종종 졸리다

3 = 자주 졸리다

Complete instrument contains 8 items. For full scale, see Johns MW (1991). A new method for measuring daytime sleepiness: the Epworth sleepiness scale. Sleep, 14(6), 540-545.

**2. Korean Version of Insomnia Severity Index (ISI)**

Example Items:

불면증 심한 정도 (최근 2주 기준)

| 증상 | 전혀없다 | 경미 | 중간 | 심함 | 극심 |
| --- | --- | --- | --- | --- | --- |
| 잠 들기 어려움 | 0 | 1 | 2 | 3 | 4 |
| 잠을 계속 자기 어려움 | 0 | 1 | 2 | 3 | 4 |

수면 패턴 만족도

| 매우 만족 |  |  |  | 매우 불만족 |
| --- | --- | --- | --- | --- |
| 0 | 1 | 2 | 3 | 4 |

Complete instrument contains 7 items. For full scale, see Bastien CH, Vallières A, Morin CM (2001). Validation of the Insomnia Severity Index as an outcome measure for insomnia research. Sleep Medicine, 2(4), 297-307.

**3. Pittsburgh Sleep Quality Index (PSQI) - Korean Version**

Example Items:

수면 습관 (지난 한 달 기준)

| 항목 | 평일 | 주말(공휴일) |
| --- | --- | --- |
| 1. 평소 몇 시에 잠자리에 들어가나요? | __시 __분 | __시 __분 |
| 2. 밤에 잠자리에서 잠이 들기까지 얼마나 걸리나요? | __시간 __분 | __시간 __분 |

수면 문제 빈도 (지난 한 달 기준)

| 문제 상황 | 한번도 없다 (0) | 한 주에 한번보다 적게 (1) | 한 주에 한두번 정도 (2) | 한 주에 세번 이상 (3) |
| --- | --- | --- | --- | --- |
| a. 30분 이내로 잠들 수 없다 | 0 | 1 | 2 | 3 |
| b. 한밤중이나 새벽에 깬다 | 0 | 1 | 2 | 3 |

Complete instrument contains 19 items across 7 components. For full scale, see Buysse DJ, Reynolds CF, Monk TH, Berman SR, Kupfer DJ (1989). The Pittsburgh Sleep Quality Index: a new instrument for psychiatric practice and research. Psychiatry Research, 28(2), 193-213.

**4. Korean Version of Beck Depression Inventory-II (BDI-II)**

Example Items:

1. 슬픔

0 = 나는 마음이 슬프지 않다

1 = 나는 자주 마음이 슬프다

2 = 나는 항상 마음이 슬프다

3 = 나는 너무 슬프고 불행해서 도저히 견딜 수가 없다

4. 즐거움을 잃어버림

0 = 나는 내가 즐겨 하는 일에 예전만큼 즐거움을 느낀다

1 = 나는 예전만큼 즐거움을 느끼지 못한다

2 = 나는 과거에 즐겼던 일에 대해 거의 즐거움을 느끼지 못한다

3 = 나는 과거에 즐겼던 일에 대해 전혀 즐거움을 느끼지 못한다

Complete instrument contains 21 items. The BDI-II is a copyrighted instrument. For full scale and usage permissions, contact Pearson Assessments or refer to Beck AT, Steer RA, Brown GK (1996). Manual for the Beck Depression Inventory-II. San Antonio, TX: Psychological Corporation.

**Instructions for Use**

Korean Version of ESS: Rate your likelihood of dozing in each situation using the 0-3 scale provided.

Korean Version of ISI: Rate the severity of your insomnia symptoms over the past 2 weeks using the 0-4 scale provided.

Korean Version of PSQI: Complete all questions regarding your sleep habits and quality over the past month.

Korean Version of BDI-II: Choose the statement that best describes how you have felt during the past 2 weeks, including today.

**Scoring Information**

ESS: Total scores range from 0-24. Scores >10 suggest excessive daytime sleepiness.

ISI: Total scores range from 0-28. Scores ≥15 suggest clinical insomnia.

PSQI: Global scores range from 0-21. Scores >5 indicate poor sleep quality.

BDI-II: Total scores range from 0-63. Scores ≥14 suggest mild to severe depression.

**Copyright Notice**

The complete versions of these instruments are protected by copyright. Researchers should obtain appropriate permissions before using full scales in their studies. The example items shown here are for illustrative purposes only and represent a small portion of each complete instrument.
